# A Cytohistologic Correlation Study of Thyroid Lesions: Evaluation of Diagnostic Accuracy and Pitfalls of Fine Needle Aspiration Cytology

**DOI:** 10.7759/cureus.55748

**Published:** 2024-03-07

**Authors:** Vidula Gowardhan, Arvind Valand

**Affiliations:** 1 Pathology, N. K. P. Salve Institute of Medical Sciences & Research Centre and Lata Mangeshkar Hospital, Nagpur, IND; 2 Pathology, Vedantaa Institute of Medical Sciences, Palghar, IND

**Keywords:** fna, surgery, histo, cyto, thyroid

## Abstract

Background

Only about 5% of palpable thyroid nodules are malignant; the rest are entirely benign. In order to reduce the number of unnecessary treatments and properly identify situations that need surgical intervention, it is essential to distinguish between benign and malignant lesions prior to surgery. There exists a “grey zone” in thyroid cytology characterized by a significant decrease in diagnostic accuracy, making it difficult to precisely classify the lesion and leading to discrepancies.

Aims and objectives

The study aims to accomplish the following objectives: (1) assess the prevalence of thyroid lesions according to age and sex; (2) evaluate the accuracy of the fine needle aspiration cytology (FNAC) in diagnosing thyroid conditions; and (3) investigate the causes of cytohistological discordance within the context of this study.

Materials and methods

In our five-year study of thyroid lesions, 125 cases were studied for cytohistological correlation. Discrepant and likened FNAs were classified according to the diagnostic findings. A review of the cytological smears and histological sections was conducted.

Results

The cytological diagnoses were correlated with histopathology in 109 cases (90.83%). A total of 11 cases (09.16%) were discrepant. There were no false positives (FPs). The causes of false negative (FN) diagnoses in our study can be attributed to geographic misses and failure to recognize dual pathologies.

Conclusion

FNA is a very precise and time-saving technique for the diagnosis and subsequent management of palpable thyroid nodules. Patients having thyroidectomies have a much higher malignant yield, and the frequency of procedures performed on the thyroid is decreased. When FNA interpretation based on strict specimen sufficiency standards is considered along with clinical and imaging findings, the occurrence of FN and FP diagnoses is expected to decrease.

## Introduction

According to reports, thyroid nodules are common clinical findings, occurring between 4% and 7% of the time in the general population [1]. Women seem to have a higher prevalence of thyroid nodules. Age, radiation exposure, and a diet high in goitrogenic substances all contribute to an increased occurrence [2]. The vast majority of thyroid nodules that can be felt are completely harmless, with just around 5% being cancerous. In order to save time and money and only operate on those lesions that really need it, it is crucial to be able to distinguish benign from malignant ones before surgery.

Presently, USG, thyroid scans, and fine needle aspiration (FNA) are the modalities utilized to evaluate thyroid nodules. Most benign thyroid nodules also appear as “cold” nodules, even though isotopically “cold” lesions are worrisome for cancer. Furthermore, malignant “warm” nodules may still exist even after thyroid-stimulating hormone suppression has occurred. Ultrasound can find nodules that are not perceptible, but it cannot tell the difference between benign and cancerous growths. Complex cystic masses and nodules that are hard to palpate are often evaluated using USG. According to the 2017 ACR TI-RADS criteria, scores of 3-5 are associated with a high risk of malignancy. USG and FNA cytology (FNAC) are equally sensitive in diagnosing malignant thyroid nodules, but FNA is more specific (90%). It is a minimally invasive method that can be used to distinguish malignant from benign lesions with a high degree of accuracy (85%) [3]. For the purpose of assessing discrete thyroid nodules, an FNA biopsy has become the procedure of choice.

FNA should be used for planning surgery for thyroid nodules and especially for follow-up with the patients [4]. Detection of thyroid malignancies at early stages is being achieved by this treatment, which is becoming more common because of its simplicity, cheap cost, and lack of serious consequences. As a result, patients are seeing improved results.

The study’s overarching goal is to determine how well thyroid FNA works and to dissect cases where cytological and histological results disagree.

## Materials and methods

This is a five-year retrospective study of thyroid lesions conducted at Grant Government Medical College, Mumbai, India. Cases involving individuals of any age or gender with thyroid enlargement who underwent both thyroid FNA and subsequent surgery were included in the study. A total of 125 cases of thyroid FNA with subsequent histopathology were studied. Five cases were excluded due to inadequacy. All the records of cytology and histology were reviewed. Trained pathologists performed all aspirates. The smears were immediately fixed in a fixative containing equal parts of 50% ethanol and 50% ether solution. Some smears were air-dried. The smears were stained with H&E, Papanicolaou, and May-Grünwald-Giemsa (MGG) stains.

The specimens were received in formalin. The type of specimen, size, weight, nodularity, capsulation, and secondary changes like hemorrhage, calcification, and cyst were noted. Two to three millimeters thick, five to 10 sections were taken and stained with H&E. When it was required, special stains were applied. All the smears as well as histopathology slides were blindly reviewed by two pathologists. Thyroid cytology was reported according to the Bethesda System 2017 as non-diagnostic or unsatisfactory (Bethesda I), benign (Bethesda II), atypia of undetermined significance or follicular lesion of undetermined significance (Bethesda III), follicular neoplasm or suspicious for a follicular neoplasm (Bethesda IV), suspicious for malignancy (Bethesda V), and malignant (Bethesda VI) [5]. Discrepant and correlated FNAs were classified according to the diagnostic findings. A review of the cases with discrepancies was done to find out if the discrepancy in diagnosis was due to histological mistakes, cytodiagnostic errors (such as overlapping criteria or misinterpretation of established criteria), FNA sample mistakes, or smears of low quality (limited by poor cellular preservation, partly hidden by blood, diluted, or having borderline cellularity).

A true positive (TP) case was one in which a definitive histological diagnosis of cancer followed a cytological diagnosis of malignancy or suspected malignancy. True negative (TN) cases were defined as those with negative cytological diagnoses and benign histological findings. A non-neoplastic lesion that often does not need surgical intervention is referred to as a false negative (FN) diagnostic when it is applied to a malignant lesion. When a non-neoplastic lesion is mistakenly diagnosed as a neoplasm and surgical removal is necessary, this is called a false positive (FP) cytological diagnosis [6].

Positive predictive value (PPV), negative predictive value (NPV), specificity, correctness of FNA to the final histology result, and sensitivity were the parameters that were calculated.

## Results

The total number of cases was 120. People in the age bracket of 31-40 years had the highest prevalence of thyroid lesions, at 37.33%. The majority of the patients were female and in their 40s and 50s (Table 1 and Table 2).

**Table 1:** Distribution of thyroid lesions by age and sex

Age (years)	Number of males	Number of females	Total (N)	% Males	% Females	Total (N%)
11-20	3	7	10	30	70	100
21-30	2	26	28	7.15	92.85	100
31-40	5	35	40	12.5	87.5	100
41-50	2	20	22	9.1	90.9	100
51-60	4	10	14	28.57	71.43	100
61-70	0	6	6	-	100	100
Total (N)	16	104	120	13.33	86.67	100

**Table 2:** Age and sex-wise incidence of various thyroid lesions including dual pathology

	Goiter	Goiter	Thyroiditis	Thyroiditis	Benign neoplasms	Benign neoplasms	Malignant neoplasms	Malignant neoplasms	Total (N)
Age (years)	Number of males	Number of females	Number of males	Number of females	Number of males	Number of females	Number of males	Number of females	
11-20	1	4	-	3	-	1	2	-	11
21-30	1	21	-	3	-	4	1	1	31
31-40	4	27	-	8	1	5	-	2	47
41-50	1	15	1	3	-	3	-	1	24
51-60	3	9	-	3	1	1	-	-	17
61-70	-	6	-	1	-	-	-	-	7
Total (N)	10	82	1	21	2	14	3	4	137

The incidence of goiter (19.70%), thyroiditis (5.83%), benign neoplasms (3.64%), and malignant neoplasms (1.45%) were found to be higher in females between 31 and 40 years of age. Most of the patients (78.34%) presented with only thyroid swelling. It was associated with signs and symptoms of hyperthyroidism in 17.5% of patients. Only one patient had a fever and pain. Classic signs and symptoms of Graves’ disease were found in only one case.

Multinodular thyroid enlargement was the most common (72.5%) presentation in this study. Among the lesions identified by cytology, nodular goiter accounted for 42.5% of cases, while simple colloid goiter was the second most prevalent. When cytology revealed a nodular goiter, the most frequent surgical procedure for thyroid lesions was a subtotal thyroidectomy. Two patients with papillary cancer had a total thyroidectomy with a radical neck dissection. The cytological diagnosis of suspicion for carcinoma resulted in total thyroidectomies.

Multinodular goiter was the most common (50%) histopathological diagnosis given. It was found to be associated with chronic thyroiditis in 9.16% of cases and with follicular adenoma in one case (0.83%).

The overall TPs were 13, the TNs were four, the FPs were two, and the FNs were two. The sensitivity was 86.66%, and the specificity was 66.66%. The PPV was 86.66%, the NPV was 66.66%, and the accuracy was 80.95%.

For malignant neoplasms, the TPs were four, and the TNs were 113. There were no FPs, but there were three FNs. The sensitivity was 57.14%, the specificity was 100%, and the accuracy was 97.5%.

The TPs for benign neoplasms were seven, the TNs were 101, the FPs were two, and the FNs were 10. The sensitivity was 41.17%, the specificity was 97.11%, and the accuracy was 90%.

## Discussion

In our five-year study of thyroid lesions, 125 cases were studied for cytohistological correlation. Five cases were excluded from the study due to inadequacy. Accordingly, the 4% insufficiency rate is lower than that of the research conducted by Gallagher et al. [7,8]. Those who were treated varied in age from 12 to 65. Maximum cases were found between 31 and 40 years of age (33.33%), with the lowest incidence in the seventh decade (5%). Females dominated the study population (86.66%) [9,10]. The female-to-male ratio was found to be 6.5:1. Most of the females (90.90%) were in the fourth decade. All the patients presented with thyroid swelling (100%). In 26 cases (21.66%), it was associated with other symptoms. A total of 17.5% of cases had associated signs and symptoms of hyperthyroidism, out of which one case showed classic clinical features of Graves’ disease. Thyroid swelling associated with pain and fever was seen in one case (acute thyroiditis). The nature of thyroid enlargement was multinodular in 87 cases (72.5%), followed by solitary nodules (15%) and diffuse enlargement (12.5%). Cytology confirmed the clinical diagnosis of toxic goiter in all three cases (100%), followed by simple goiter. It helped to detect five cases (4.16%) of malignancies that were not suspected clinically (Table 3).

**Table 3:** Clinico-cytological correlation of thyroid lesions

Clinical diagnosis	Colloid goiter	Nodular goiter	Graves’ disease	Acute thyroiditis	Chronic thyroiditis	Hashimoto’s thyroiditis	Follicular neoplasm	Suspicious of carcinoma	Papillary carcinoma	Total (N)
Thyroid cyst	1	1								2
Goiter	42	37		1	3	4	8	3	1	99
Toxic goiter		1	1				1			3
Solitary thyroid nodule		12					3		1	16
Total (N)	43	51	1	1	3	4	12	3	2	120

The cytological diagnosis of suspicious carcinoma (Bethesda V) resulted in total thyroidectomies. The total thyroidectomy and radical neck dissection were carried out for the cytological diagnosis of papillary carcinoma (Bethesda VI). The benign cytological diagnoses resulted in subtotal thyroidectomy in 75 patients (62.5%), followed by other surgeries. A total of 87 out of 94 cases (92.55%) of goiter were diagnosed on cytology. Associated lesions like adenomatous change in seven cases and chronic thyroiditis in nine cases were missed during sampling (Table 4).

**Table 4:** Cyto-histo correlation of thyroid lesions FNA, fine needle aspiration

FNA diagnosis	Number of correlated cases	% of correlated cases	Number of discrepant cases	% of discrepant cases	Total number of cases (N)
Bethesda II (Goiter)	87	92.55	7	7.45	94
Bethesda II (thyroiditis)	8	88.89	1	11.11	9
Bethesda IV (benign neoplasm)	9	69.23	4	30.77	12
Bethesda VI (malignant neoplasm)	5	100	0	-	5
Total number of cases (N)	109	90.83	11	9.16%	120

The cytologic criteria for adenomatous nodule and follicular adenoma overlap, leading to the misinterpretation of follicular adenoma (Bethesda IV) as goiter (Bethesda II) in three instances when it was coupled with chronic thyroiditis. This occurred in six cases in total. A repetitive microfollicular pattern was not seen in the moderately cellular cytologic smears, which showed flat sheets of follicular cells with limited nuclear overlap. Moreover, a colloid was seen in the backdrop. Histologically, these cases were diagnosed as follicular adenomas.

**Figure 1:**
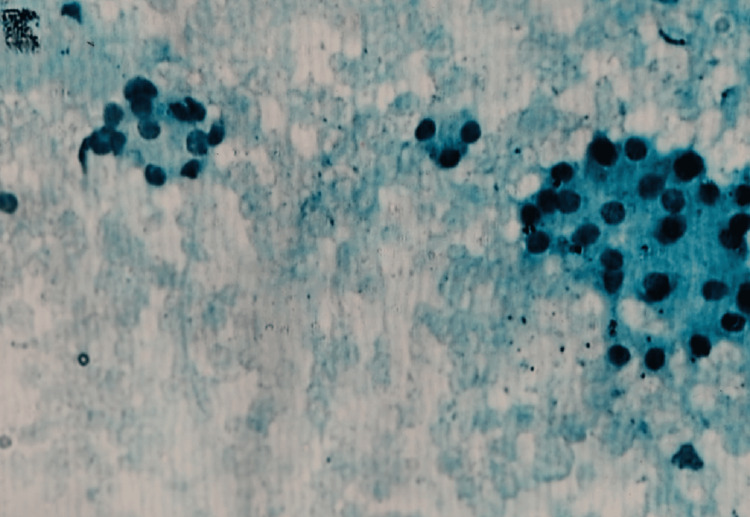
Follicular neoplasm: photomicrograph of FNA smear showing the microfollicular arrangement of follicular cells (Papanicolaou stain 20X) FNA, fine needle aspiration

The cytomorphological overlap between these two entities is well recognized (Figure 1).

A follicular neoplasm is likely when syncytial pieces are present and a microfollicular pattern is seen; nevertheless, this may occur in 10-25% of nodular goiter cases. In terms of background colloid, these entities constitute a diminishing order, while in terms of cellularity and microfollicles, they form an increasing order, according to Bussenier and Oertel’s findings [11].

**Figure 2:**
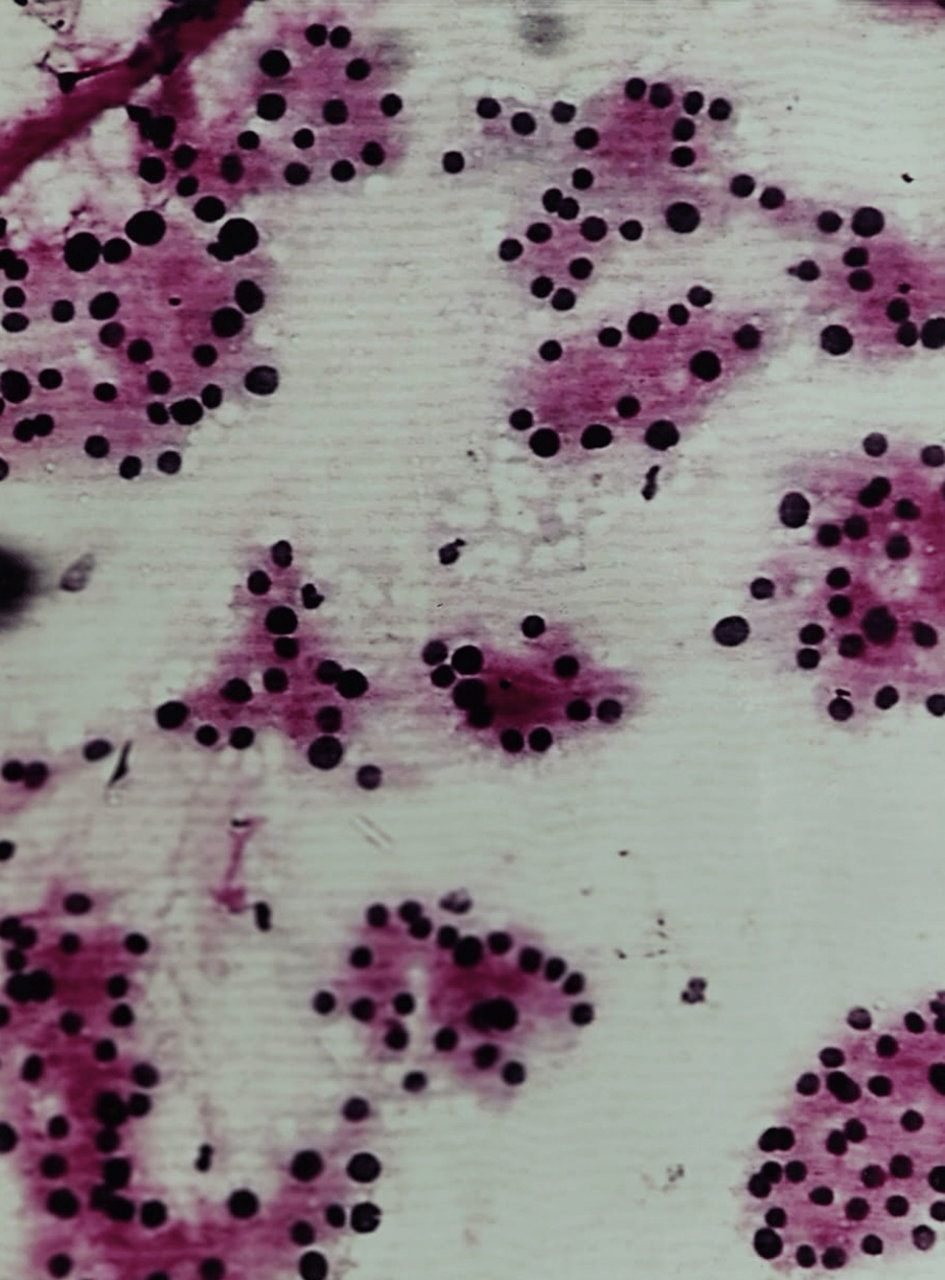
Graves’ disease: photomicrograph of FNA smear showing follicular cells displaying “fire flares” (H&E stain 20X) FNA, fine needle aspiration

There were eight cases of thyroiditis (including one case of Graves’ disease). The patient with Graves’ disease presented with classic signs and symptoms along with laboratory evidence of hyperthyroidism. The cytologic smears revealed moderate cellularity of follicular cells in clusters and follicles against a hemorrhagic background. There was evidence of nuclear pleomorphism and marginal vacuoles (Figure 2).

The diagnosis of Graves’ disease was favored over that of toxic goiter based on clinical and cytological features.

One case of acute thyroiditis was accurately diagnosed considering the symptomatology (fever, pain, and tenderness) and cytology. The smears revealed neutrophils against a dirty, necrotic background. Three cases of chronic thyroiditis were diagnosed on cytology based on the presence of follicular cells along with a moderate number of lymphocytes and scant colloids. In two cases, the associated nodular goiter was missing during sampling. One case was diagnosed in histology as chronic fibrosing thyroiditis.

When comparing inflammatory goiter with thyroid neoplasms, Das et al. found that the former had a much greater number of lymphocytes, plasma cells, lymphohistiocytic aggregates, epithelioid cells, and giant cells. It is possible to mistake papillary cancer for thyroiditis due to the lymphocytic infiltration [12].

**Figure 3:**
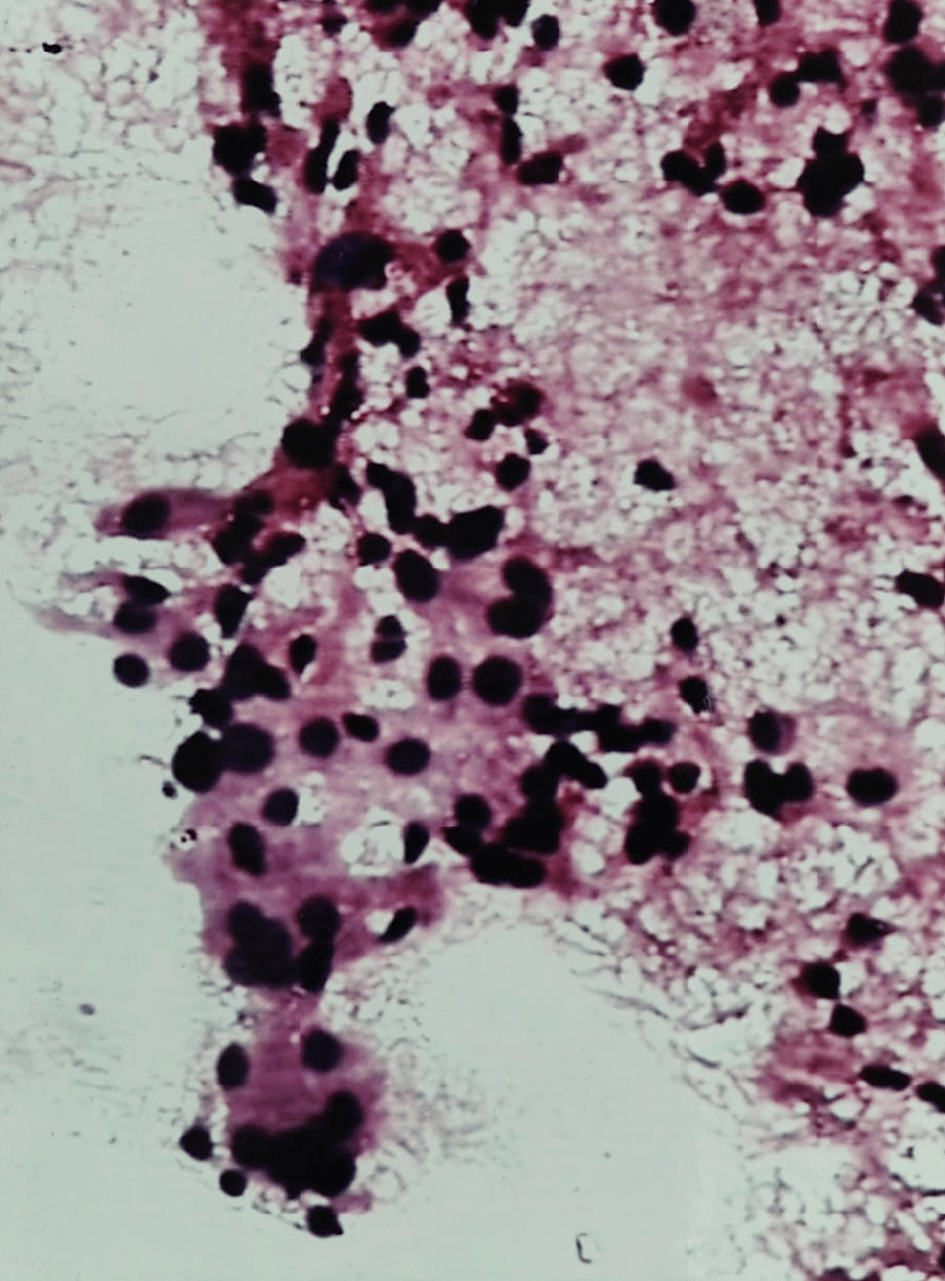
Hashimoto’s thyroiditis: photomicrograph of FNA smear showing Hürthle cells, lymphocytes, and scanty colloid (H&E stain 10X) FNA, fine needle aspiration

All three cases of Hashimoto’s thyroiditis were accurately diagnosed by cytology. Nguyen has reported a cytologic diagnostic accuracy rate of 92% for Hashimoto’s thyroiditis [13]. The smears were moderately cellular and showed the presence of Hürthle cells along with lymphocytes, scant colloid, and occasional giant cells (Figure 3).

**Figure 4:**
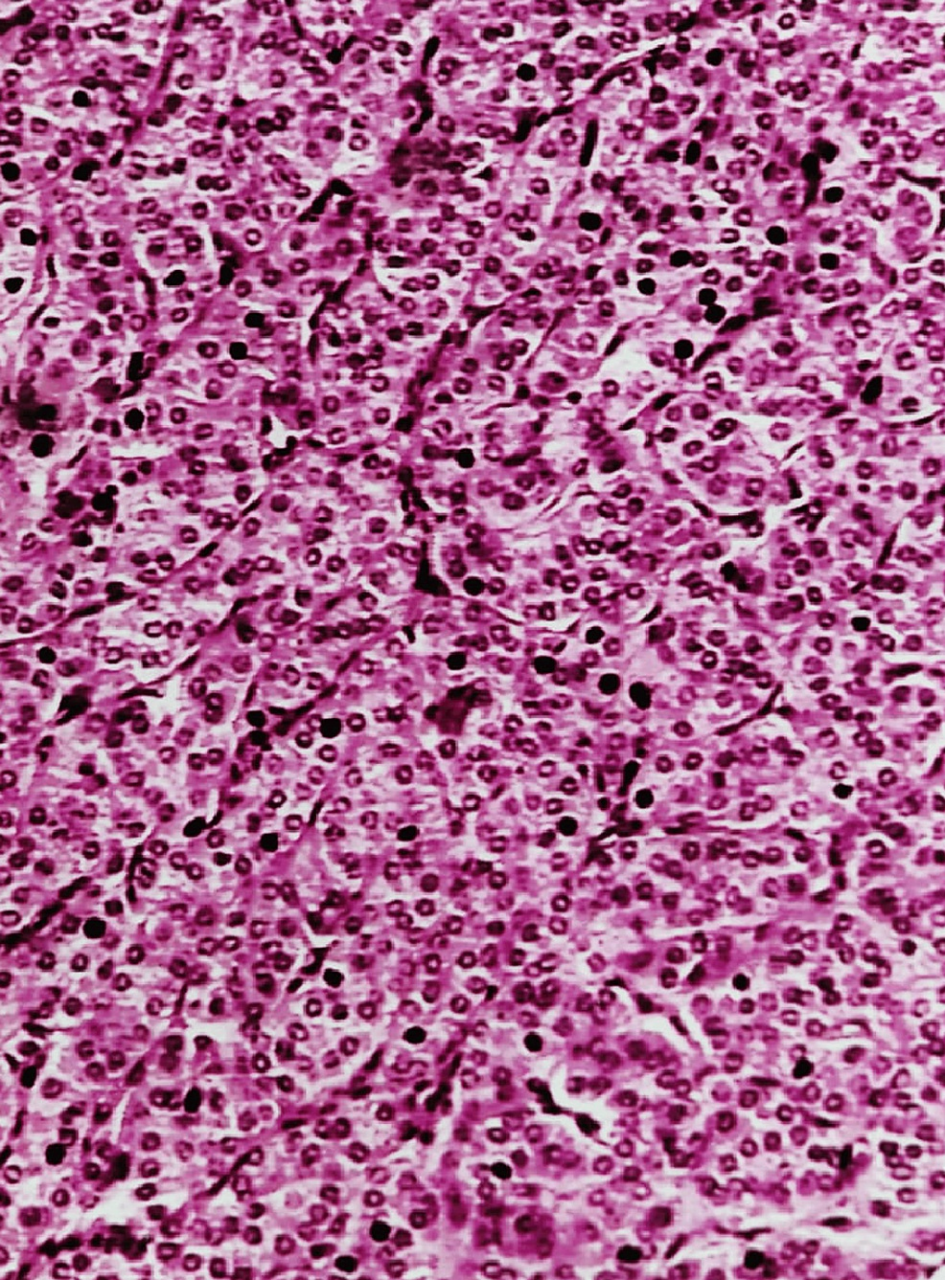
Hürthle cell adenoma: photomicrograph showing the follicular arrangement of Hürthle cells (H&E stain 20X)

One case of follicular (Hürthle cell) adenoma (Bethesda IV) was misdiagnosed as Hashimoto’s thyroiditis (Bethesda II) on cytology. The smears were cellular and revealed a few lymphocytes and loose clusters of Hürthle cells with the attempt to form follicles at places. Histology showed a typical Hürthle cell adenoma (Figure 4).

**Figure 5:**
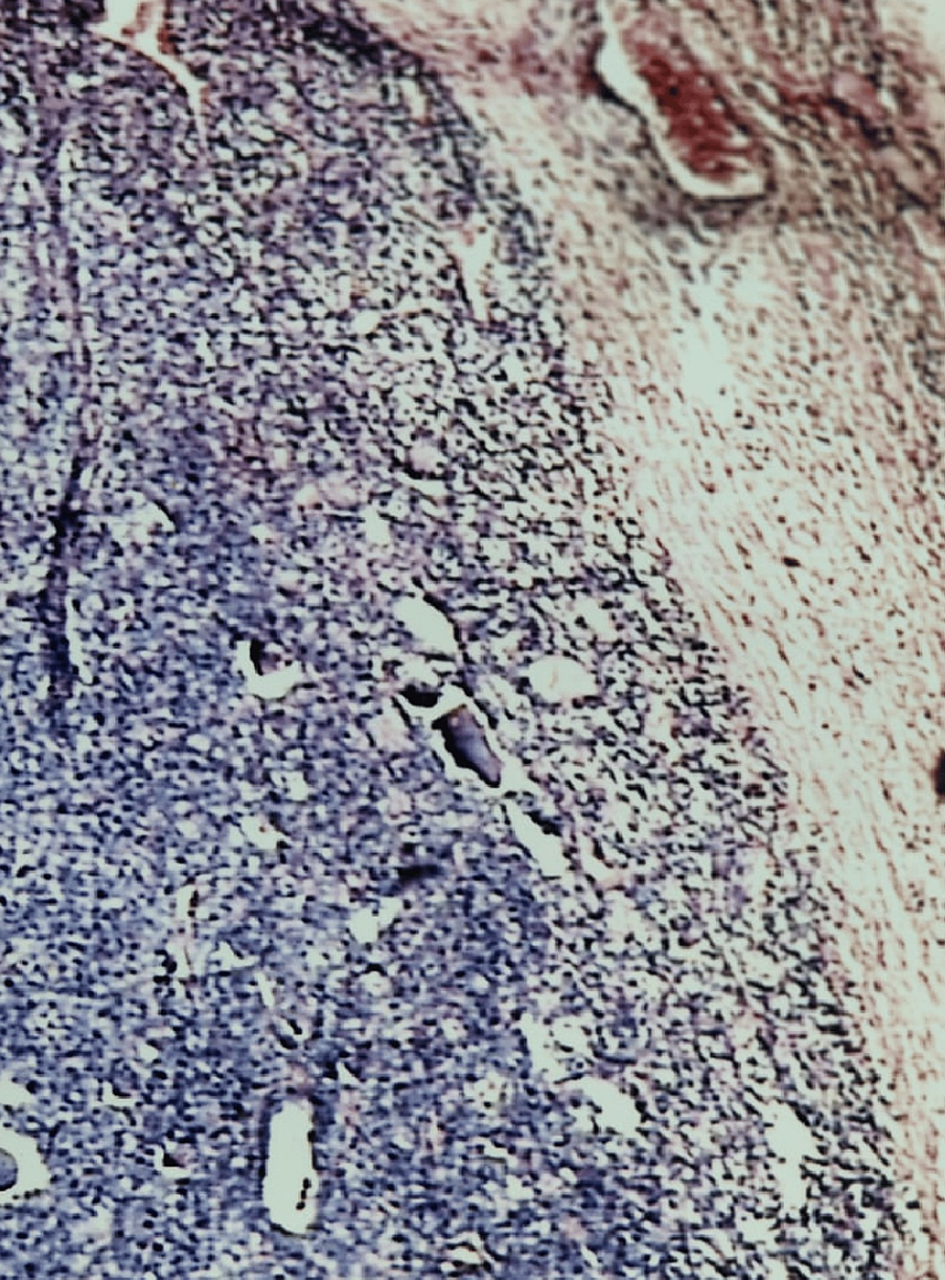
Follicular adenoma: photomicrograph showing a well-encapsulated tumor with a microfollicular arrangement (H&E stain 4X)

A total of 16 benign neoplasms were identified in histology. One was a Hürthle cell adenoma, and the remaining were follicular adenomas (Figure 5).

Among the 12 follicular neoplasms diagnosed on cytology, nine were follicular adenomas, and one was follicular carcinoma on histology. The two FPs included multinodular goiters with adenomatous change in one case and chronic thyroiditis in another. The FP rate was 16.66%. Among 16 follicular adenomas diagnosed in histology, nine were diagnosed cytologically, one was misdiagnosed as Hashimoto’s thyroiditis, and six as nodular goiters. The FN rate was 43.75%.

It was determined that one instance of pure follicular malignancy was a follicular neoplasm based on cytology results. According to Löwhagen and Sprenger, follicular adenomas and follicular carcinomas have similar cytologic characteristics; hence, they suggest using the umbrella term “follicular neoplasms” to describe them both [14]. The final diagnosis should be derived from histological evidence of vascular invasion and capsular invasion. The study by Deshpande et al. also supports this view [15].

Among follicular adenomas (Bethesda IV), 14 were seen in females and two in males. The maximum incidence was found in the fourth decade. Three follicular adenomas were associated with chronic thyroiditis, and one was associated with multinodular goiter. All four cases were misdiagnosed as nodular goiter (Bethesda II) on cytology, an error of no particular significance to the patient. Based on the cytology report, six patients underwent hemithyroidectomy, four subtotal thyroidectomy, and two lobectomies.

**Figure 6:**
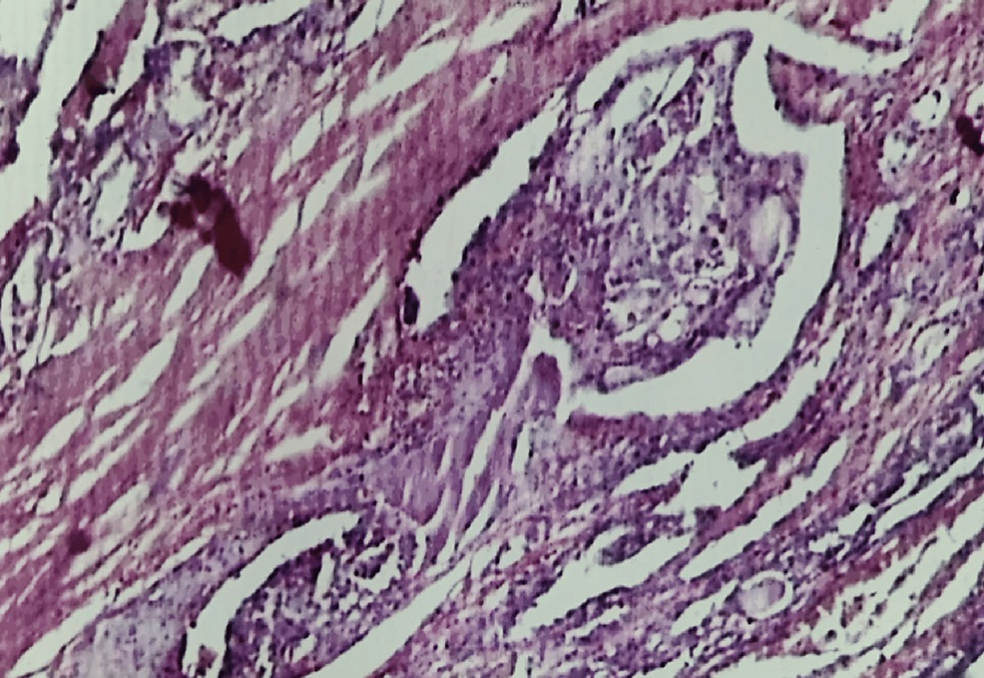
Follicular carcinoma: photomicrograph showing vascular invasion (H&E stain 40X)

Among malignancies (Bethesda VI), there were six papillary carcinomas and one follicular carcinoma. Six were accurately diagnosed with cytology (85.71%). A male patient, aged 30, who had one thyroid nodule, was found to have follicular carcinoma. Due to their diagnostic cytologic overlap, it was determined to be a follicular neoplasm in cytology and was ruled a genuine positive. There were discohesive clusters, syncytial sheets, and fairly cellular follicular cells in the smear. There was evidence of nuclear pleomorphism. The background revealed a hemorrhage with scant colloid. Vascular invasion was demonstrated in histology (Figure 6).

Based on the cytology report, the patient underwent a subtotal thyroidectomy. The literature reports that between 15% and 22% of nodules classified as follicular neoplasms by cytology end up being malignant [16]. Out of 219 individuals who were suspected of having follicular neoplasms, 16% were found to have carcinoma, according to the pathology report [17]. Malignant lesions are present in only around 14% of lesions cytologically identified as follicular neoplasms, according to Gharib’s findings [18].

**Figure 7:**
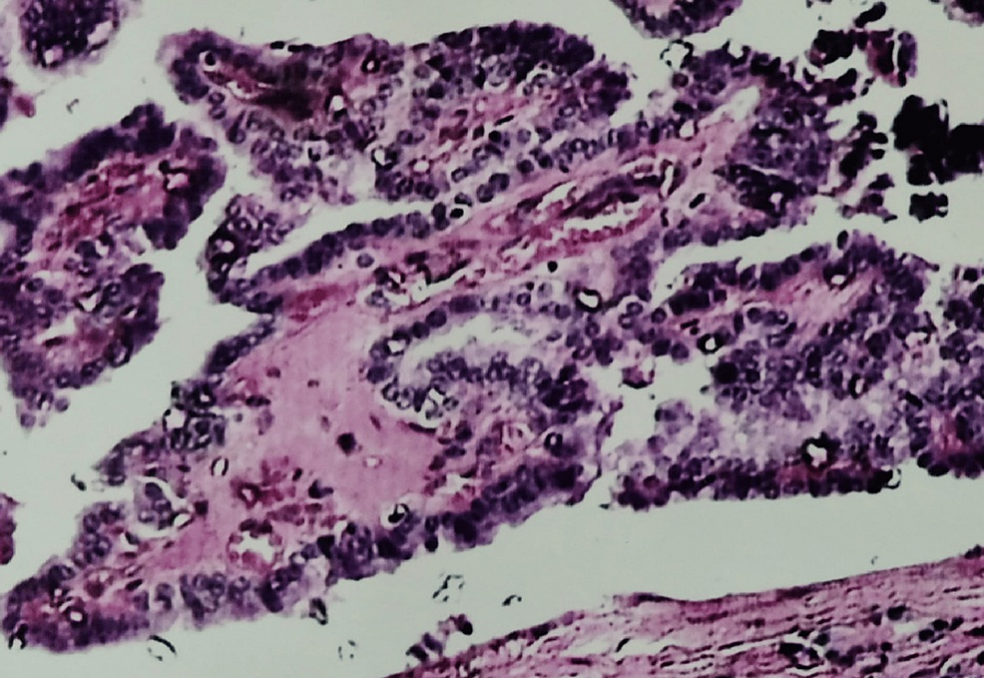
Papillary carcinoma: photomicrograph showing papillae with a central fibrovascular core (H&E stain 10X)

Two cases of papillary carcinoma were seen in males in the second decade. The remaining four cases were seen in females in the third decade (one case), fourth decade (two cases), and fifth decade (one case). Malignancy was not suspected in any of these patients, who presented with solitary thyroid nodules. All were euthyroid, except for two patients who had hypothyroidism. As far as we can tell, three of the instances were papillary carcinoma. The smears were highly cellular, exhibiting loosely cohesive clusters, papillary fragments, syncytial sheets, and dissociated cells against a hemorrhagic background. The nuclei were mildly pleomorphic, with the presence of grooves in some of the nuclei. The histological sections in two cases revealed papillary fronds with a fibrovascular core (Figure 7).

**Figure 8:**
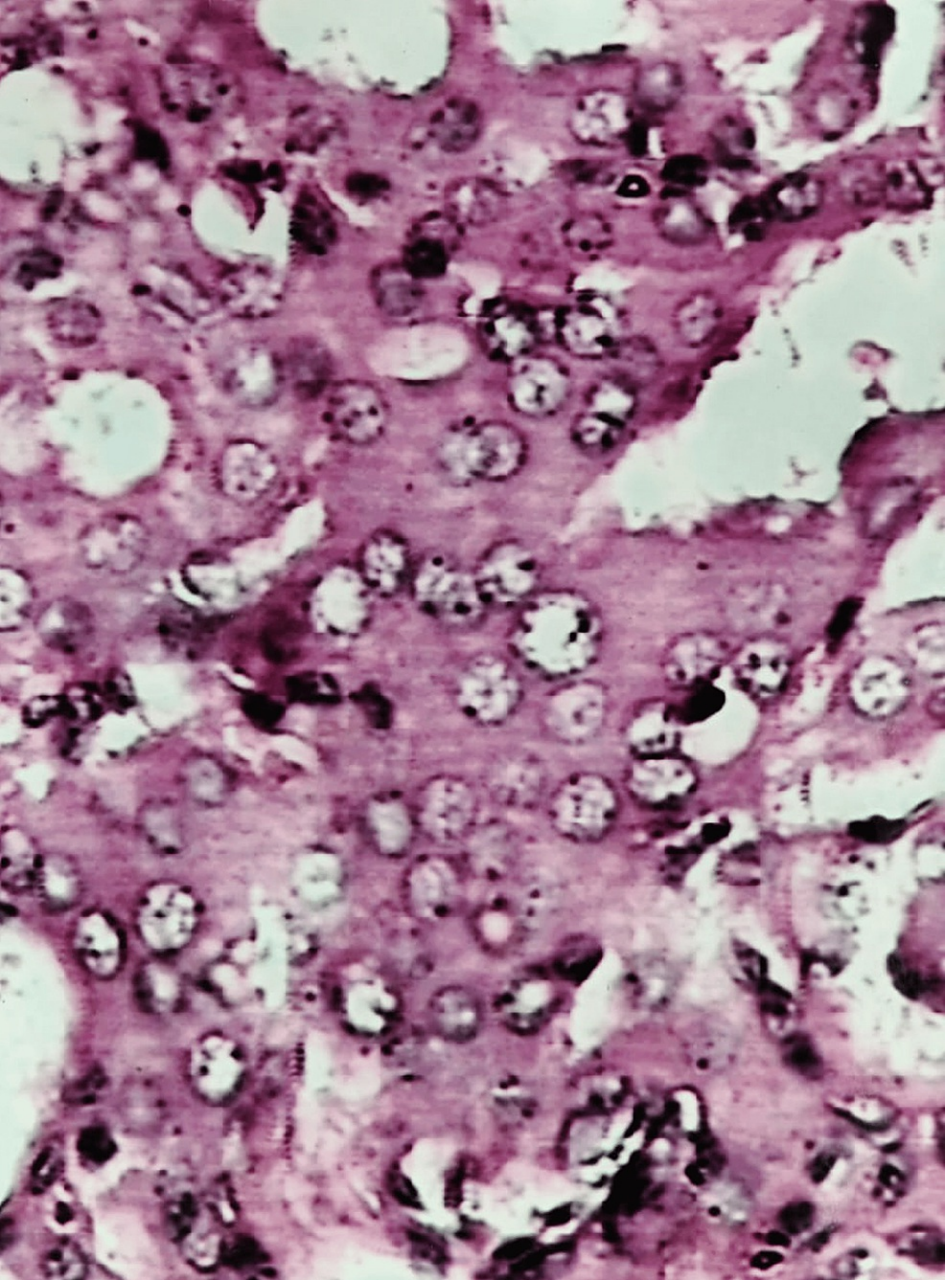
FPVC: photomicrograph showing optically clear nuclei (H&E stain 10X) FVPC, follicular variant of papillary carcinoma

The classic nuclear characteristics of papillary cancer were seen in the cells. To differentiate papillary cancer from benign lesions, Prabhu and Umashankar have concluded a diagnostic score based on parameters like colloid, papillae, nuclear crowding, nuclear grooving, nuclear enlargement, oval nucleus, and nuclear clearing as >7 for papillary thyroid cancer [19]. A follicular form of papillary cancer was found in the third patient (Figure 8).

Follicular variant of papillary carcinoma (FVPC) is a name provided by Lindsay, who states that these tumors look like the more prevalent types of papillary carcinoma but often lack papillary features [20]. In cytologic preparations of FVPC, papillary fronds have been discovered. Two studies, one by Das et al. and the other by Leung et al., detected them in around half of the patients [12,21]. As compared to follicular variations, typical papillary carcinoma included more papillary structures, monolayered sheets, and nuclear grooves. Follicle-like features, dusty chromatin, and intranuclear inclusions were more often seen in typical carcinoma. In smears stained with H&E, Francis et al. discovered nuclear grooves, which are a helpful diagnostic tool for papillary thyroid carcinoma (PTC) [22]. However, in smears stained with the MGG stain, the reliability of this finding is diminished. It has to be differentiated from noninvasive follicular thyroid neoplasms with papillary-like nuclear features (NIFTPs). Since NIFTP shows subtle or focal nuclear features of PTC with a follicular morphology, the differential diagnosis between NIFTP and follicular adenoma is difficult. The diagnostic criteria for NIFTP include the absence of papillae, strict cytomorphonuclear features (typically more subtle than the follicular variant of PTC and mainly localized to the periphery of the neoplasm), no psammoma bodies, no necrosis, and no more than three mitoses per 10 high-power fields [23].

The other two cases of papillary carcinoma were accurately diagnosed by cytology based on papillary fragments and nuclear features, which include fine chromatin, grooves (100%), and intranuclear inclusions (100%). Psammoma bodies could not be found in any of the cases. One case of papillary carcinoma (Bethesda VI) was underdiagnosed as nodular goiter (Bethesda II). Histology revealed goitrous areas in the tissue surrounding the carcinomatous area, which were missed during sampling. The cytologic diagnosis of papillary carcinoma was shown by Ko et al. [24] to have a 100% predictive value. Many nuclear changes and a fine chromatin architecture are characteristics of neoplastic non-papillary carcinoma lesions, as reported by Mai et al. [25]. Nodular goiter, follicular adenoma, and papillary carcinoma are sometimes difficult to distinguish cytopathologically due to these abnormalities.

The limitations of this five-year research include a small sample size and the unexpected absence of cases involving Hürthle cell carcinoma, medullary carcinoma, or anaplastic carcinoma. The cytological diagnoses were correlated with histopathology in 109 cases (90.83%). A total of 11 cases (09.16%) were discrepant. This correlation rate is slightly higher as compared to other studies. Mitra et al. achieved a correlation rate of 75% [26], whereas it was 88% in a study by Bakhos et al. [27]. The correlation for malignant neoplasms was 100%, followed by goiters (92.55%) (Table 4).

The accuracy of FNA in diagnosing malignant conditions was 97.5%, with a sensitivity of 71.42% and a specificity of 100%. Our sensitivity for benign neoplasms was 81.81%. When neoplasms, in general, were considered, sensitivity was 87.5%, specificity was 98.07%, and accuracy was 96.66%. The above indices agree with Abu-Salem [28], Amrikachi et al. [29], and Baloch et al. [30] (Table 5).

**Table 5:** Comparison with other studies

Series	Total number of aspirates	Accuracy	Sensitivity	Specificity
Afroze et al. [6]	170	94.58%	61.90%	99.31%
Čáp et al. [31]	2,492	75%	86%	74%
Bakhos et al. [27]	625	88%	93%	96%
Ko et al. [24]	1,613	84.4%	78.4%	98.2%
Abu-Salem [28]	520	94%	93%	99%
Baloch et al. [30]	662	-	92%	84%
Amrikachi et al. [29]	6,226	-	93%	96%
This study	120	97.5%	71.42%	100%

There is a wide range of specificity (74%) and sensitivity (61.9-100%) in the reported series [24,27-31]. The study done by Čáp et al. showed low specificity and PPV because the results were divided into only two categories, which were indications for surgery or not [31]. Variation among cytopathologists in how they handle the “suspicious” category and define FPs and FNs is the major cause of such a broad range of sensitivity and specificity. Nothing came back as a FP. A FP rate of 1-8% has been recorded by several authors [6,24,29]. Incorrectly diagnosing follicular neoplasms is the leading cause of FP results [24]. A few FP reports are acceptable if one does not want to miss any of the malignancies.

Among all neoplasms, there were three FNs. The reported incidence in the literature varies between 1% and 16% [6,27,29]. There was less than a 1% FN rate reported by Amrikachi et al. [29]. A far greater unsatisfactory FN rate of 42% was observed in the publication by Giard and Hermans [32].

Causes of FN diagnoses in our study can be attributed to geographic misses and follicular neoplasm, where it is difficult to differentiate between adenomas and carcinomas, and failure to recognize dual pathologies.

## Conclusions

The highest prevalence of thyroid nodules was found in females in their 40s. This study has highlighted FNA as a highly accurate and effective technique for diagnosing and treating palpable thyroid nodules. The number of incorrect diagnoses, both positive and negative, is minimal. When it comes to diagnosing nodular goiter, thyroiditis, and carcinoma, particularly papillary carcinoma, it is quite specific. Evaluating FNA’s usefulness in identifying follicular lesions is challenging. The primary issue is the lack of clear differentiation between benign lesions like follicular adenomatous goiter or follicular carcinoma and malignant lesions like follicular carcinoma or a FVPC. Thyroid FNA is still an effective screening technique, even if it has its limits. Overlapping cytologic characteristics, especially among hyperplastic nodules, follicular neoplasms, and follicular forms of papillary carcinoma, make it impossible to avoid indeterminate FNA findings and cytodiagnostic mistakes. When FNA interpretation based on strict specimen sufficiency standards is considered along with clinical and imaging findings, the occurrence of FN and FP diagnoses is expected to decrease.

It is important to closely monitor patients whose cytologic results are benign. In very rare cases, a patient may have a FN result. So, if a patient is a surgical candidate, it is important to remove clinically worrisome lesions, even if the cytology results are benign.
